# The pathway to establishing and implementing opioid agonist therapy (OAT) for people who inject drugs in Nairobi, Kenya

**DOI:** 10.3389/fpubh.2026.1717635

**Published:** 2026-08-03

**Authors:** Emily Koech, Christopher Welsh, Mercy Karanja, Jebet Boit, Rebecca Wangusi, Patrick Awuor, Oscar Munyao, Tina Masai, Sam Wafula, Caroline Ngunu, Obwiri Kenyatta, Immaculate Mutisya, Jonathan Mwangi, Beatrice Gichuhi, Ansley Lyn, Kristen Stafford, Taylor Lascko, Geoffrey Ndichu, Caroline Ng'eno

**Affiliations:** 1 Center for International Health, Education, and Biosecurity (Ciheb)-Kenya, Nairobi, Kenya; 2 Division of Addiction Research and Treatment, Department of Psychiatry, University of Maryland School of Medicine, Baltimore, MD, United States; 3 Department of Medical Services, Ministry of Health, Nairobi, Kenya; 4 National AIDS and STIs Control Programme, Ministry of Health, Nairobi, Kenya; 5 Nairobi Metropolitan Services, Department of Health, Nairobi, Kenya; 6 Division of Global HIV and TB, Global Health Center, U.S. Centers for Disease Control and Prevention, Nairobi, Kenya; 7 Division of Global Health Sciences, Department of Epidemiology and Public Health, University of Maryland School of Medicine, Baltimore, MD, United States; 8 Institute of Human Virology, University of Maryland School of Medicine, Baltimore, MD, United States; 9 Center for International Health, Education, and Biosecurity, University of Maryland School of Medicine, Baltimore, MD, United States

**Keywords:** drugs, heroin, Kenya, methadone, opioid agonist therapy

## Abstract

Sub–Saharan Africa (SSA) is experiencing a burgeoning population of people who inject heroin. This has created an urgent need for the scale-up of comprehensive harm reduction services including medication for opioid use disorders. The rise in illicit drug use threatens the gains made in the fight against HIV in this high HIV burden region. People who inject drugs (PWID) have an increased risk of HIV acquisition and transmission due to factors such as needle sharing and engaging in high-risk sexual behaviors. Estimates show that PWID have a prevalence of HIV of 18.7%-nearly four times the prevalence in the general population in Kenya. To address this, Kenya adopted the comprehensive harm reduction package of services for PWIDs and in 2013, began preparation for the provision of Opioid Agonist Therapy (OAT) with the first clinic opening in 2014 at Mathari National Teaching and Referral Hospital (MNTRH). This paper describes the process and collaborative steps taken in establishing the OAT program in Kenya. It shares implementation achievements and lessons learned. Since the inception of OAT services in Mathari and Ngara clinics in Nairobi, over 80 healthcare providers were trained on the provision of OAT services between 2014 and 2023, and 3,354 individuals were initiated on OAT. The OAT program has expanded to include mobile OAT dispensing and satellite dispensing clinics, and in future, may include the use of take-home doses of Methadone and Buprenorphine and provide injectable long-acting Buprenorphine for increased uptake and continuity in treatment.

## Highlights

- The initiation of the OAT program in Kenya was driven by the disproportionately high HIV prevalence among PWID (estimated at 18.7%) with sub-optimal access to HIV services.
- The successful implementation of the OAT program in Kenya was facilitated by strong government leadership and the structured development of standardized guidelines and standard operating procedures to guide program implementation.
- The engagement of key stakeholders, particularly community based and civil society organizations, was crucial for the success of the program through efforts toward demand creation, follow-up and support for clients on OAT.

## Introduction

Opioid Use Disorder (OUD) is a growing global health concern caused by use of opiates for clinical (prescription) and non-clinical use. In 2023, approximately 316 million people worldwide used opiates, marking a 28% increase over the previous decade. Of these, 61 million were non-medical opioid users, and 64 million had a drug use disorder (1). While data is scanty in sub– Saharan Africa, the upsurge in opioid use, misuse and OUD in Africa is concerning and requires urgent attention (2, 3). Kenya emerged as a drug trafficking route in the 1980s with the entry of “brown sugar” heroin into the market, and in the 1990s a water-soluble form of heroin known as “white crest” slowly replaced it (4, 5). Since then, the country has experienced an upsurge in the number of PWID which were estimated at 18,000 in Kenya in 2012 (6). A rapid situational assessment of the status of drug and substance use in Kenya in 2007, showed an increase in the lifetime use of heroin among persons aged 15–65 years from 0.4% in 2007 to 0.7% in 2012 (7).

Sub Saharan Africa has grappled with the highest burden of HIV in the world, with 65% of people living with HIV contributing an estimated 50 and 70% of annual new HIV infections and AIDS related mortality respectively (8). The past decade has seen the implementation of multifaceted approaches toward controlling the epidemic, adopting biomedical, behavioral, and structural interventions in HIV programming. This has resulted in a significant reduction in HIV incidence from 0.35% in 2010 to 0.14% in 2019 and a 48% reduction in HIV-related deaths during the same period (8). Despite this, the HIV prevalence among PWID has remained unacceptably high putting at risk the gains made to control the epidemic in the region (9). The estimated HIV prevalence among PWID in Nairobi was 18.7% (10), nearly four times higher than that in the general population (11). Further, the increased risk for HIV, hepatitis B, and hepatitis C transmission among opioid users has persisted over the years due to the practice of sharing injecting equipment, multiple injections in a day, injections in unhygienic environments, and unsafe sexual practices. These has created a bridge for HIV transmission to the wider population (5, 12, 13). Over 75% of PWID injected daily, of whom 35% inject at least three times a day, and 67% engage in a high-risk injecting practice, such as sharing injecting equipment (10, 14). The sub-optimal access to the comprehensive HIV prevention services including treatments for OUD is a threat to the control of HIV as a public health threat in Kenya and in countries with a growing population of PWIDs.

Management of substance use disorder in Kenya prior to establishment of OAT programs was mostly focused on alcohol use disorders. It was restricted to private rehabilitation facilities and government-owned mental health facilities. Beginning in the mid 1990′s and expanding through the early 2000′s, community-based organizations (CBO) and civil-society organizations (CSO) such as The Omari Project, Reach-out Center Trust, Support for Addictions Prevention and Treatment in Africa, Nairobi Outreach Services Trust, MDM and the Muslim Education and Welfare Association, began providing support and harm reduction services for substance users. With support from the Kenya AIDS NGOs Consortium (KANCO), the CBOs and CSOs pioneered the provision of needle and syringe program (NSP) in collaboration with Kenya's Ministry of Health, the National Authority for the Campaign Against Alcohol and Drug Use (NACADA), United Nations Office on Drugs and Crime (UNODC) under the funding from Global Fund to Fight AIDS, Tuberculosis and Malaria (GFATM) in 2008.

Kenya's Ministry of Health (MoH) developed standard operating procedures (SOPs) to guide the countrywide NSP program in 2013 with the aim of promoting safe injecting practices among PWID. The MoH subsequently developed guidelines and SOPs that guided the provision of structured biomedical, behavioral and structural services for this population subset (14) including the National Standard Operation Procedures for Establishing and Operating Drop-In Centers for Key Populations in Kenya 2016. These SOPs guided the establishment of community spaces for PWIDs to enhance access to health services, information and support (15).

Treatment of OUD previously focused on symptomatic management using medications such as clonidine, combined with psychotherapy as medically assisted withdrawal management (“detoxification”). However, this resulted in high dropouts and relapse rates (16, 17). Opioid Agonist Therapy (OAT) has been shown to be effective in reducing injection drug use by up to 40–60% and reducing the risk for HIV acquisition and transmission (18). This can reduce the risk of death by 50% for those on treatment (19). The OAT has also been shown to reduce HIV risk behaviors, such as multiple sexual partners and unprotected sexual encounters (31). Despite this evidence, OAT in sub-Saharan Africa has remained inaccessible, with minimal treatment programs. Recent studies show that only seven countries in Africa have OAT programmes (20).

This paper described the pathway through which the OAT program was established and implemented in Kenya. Further, the paper shares key achievements in the roll-out of OAT programs in Nairobi and lessons learned for future programming. This description is critical for learning and replication of this model in similar settings/countries especially in Africa where such programs remain non-existent or still at infancy stages of development.

### Introduction of opioid agonist therapy (OAT) in Kenya

The journey toward the establishment of clinics providing OAT in Kenya was triggered by a heroin shortage in the coastal region between December 2010 and March 2011, precipitated by an increase in crackdown and arrests of drug traffickers (21). This resulted in an upsurge of heroin users who visited hospitals with severe withdrawal symptoms. Part of the government's medical response was to use a codeine detoxification protocol to manage opioid withdrawal. However, when heroin became available on the street, most of the clients relapsed, resulting in an increase in drug overdoses due to reduced tolerance that had developed during the shortage (21).

In 2012 the government saw an urgent need to introduce other highly effective evidence-based interventions for the management of OUD. The aim of these initiatives was to provide comprehensive services to PWID to decrease their risk of HIV acquisition and transmission among various stakeholders were engaged including but not limited to the Pharmacy and Poisons Board (PPB), CBOs, CSOs, religious organizations, communities and bilateral and multilateral partners and donors. The donors included United Nations Office on Drugs and Crime (UNODC), CDC and United States Agency for International Development (USAID). Consequently, in 2014, the National Guidelines for HIV/STI Programming with Key Populations 2014 were developed (14). These guidelines were adopted from the World Health Organization, UNODC and UNAIDS technical guide (22). These guidelines provided a nine-point comprehensive package of evidence-based intervention to reduce harms associated with injecting drug use namely: 1) Needle and syringe programme (NSP) 2) Opioid Agonist Therapy (OAT) and other evidence-based drug dependence treatment; 3) HIV testing and counseling; 4) Antiretroviral therapy; 5) Prevention and treatment of STIs; 6) Condom programmes for PWID and their sexual partners; 7) Targeted information, education, and communication to PWID and their sexual partners; 8) Prevention, vaccination, diagnosis, and treatment for viral hepatitis; and 9) Prevention, diagnosis, and treatment of TB. The Ministry later added a tenth component on overdose management through community distribution of naloxone.

#### Cross learning sessions

In June 2011, a regional workshop on HIV and Drug Use was held in Nairobi, with participants from government, CSOs, and multilateral agency representatives from Kenya, Tanzania, Nigeria, Seychelles, Mauritius, Madagascar, Mozambique, and South Africa. This provided a forum for learning across countries e.g., Mauritius and Tanzania, which had introduced OAT programs in 2006 and 2011, respectively shared their experiences. In 2013, a core team from Kenya's MoH and the University of Maryland, Baltimore (UMB) Kenya program, with funding from PEPFAR through CDC, visited a clinic providing OAT in Tanzania for a first-hand experience on the operations of the clinic. Following this visit, the team developed a standardized tool to guide the assessment of health facilities in preparation for provision of OAT services.

#### Governance/guideline development

UMB and the Kenyan team, with funding from PEPFAR through CDC, and other stakeholders including UNODC, provided technical assistance to MoH- NASCOP to develop the first-ever National Implementation Guidelines for Medically Assisted Therapy for People with Opioid Use Disorders and updated this in 2021 (23). This marked the beginning of the process toward establishing OAT clinics in Kenya. The objectives of the OAT program were to 1). Reduce morbidity and mortality associated with illicit opioid use 2). Prevent transmission of HIV and other blood borne infections 3). Reduce drug-related crime 4). Provide psychosocial support and 5). Reduce stigma and create an enabling environment to support access to healthcare, adherence to treatment, and social reintegration. Kenya used Methadone as OAT which is classified as a controlled substance as per the Narcotic Drugs and Psychotropic Substances Control) Act (24).

Key processes in the establishment of OAT services included: 1). The development of the National Guidelines for HIV/STI Programming with Key Populations in 2014 (14); 2) Development of the national OAT roll-out and scale-up plans recognizing the role of CBOs/CSOs in the identification, preparation and follow-up of PWIDs initiated on OAT; 3) Formation of the NASCOP-led multisector committee to conduct site assessments to determine the appropriate facilities to establish the OAT clinics; 4) Development of the National Protocol for Treatment of Substance Use Disorders in Kenya, 2017 (25); 5) Development of training curricular on OAT designed with a trainer of trainers' model to rapidly cascade the training to multiple healthcare providers (HCPs); 6) Development of monitoring and evaluation tools for data collection and reporting with clear indicators to monitor quality of the services (26, 27); 7) Engagement of PPB to ensure compliance of the clinics with regulatory standards on storage, prescription and dispensing of controlled substances like opioids for security, accountability and safe dispensing. PPB further approved the use of *MethaMeasure*, an automated Methadone dispensing system (software and dispensing tool) to dispense the drug. The software allows for integration with patient biometrics to ensure that the correct client receives the correct doses. Other factors considered included patient safety, usability, speed, cost, and interoperability with other systems already in existence; 8) Engagement of the law enforcement officers, particularly the Kenya Police from program inception through training and sensitization to educate them on the relevance of the clinic, address the widespread stigma and arrest of PWID who are often viewed as criminals and support referrals of those arrested and on treatment to continue to get their daily medication; and 9) Oversight of OAT scale-up by NASCOP with day-to-day technical support by UMB and NCC. KEMSA was tasked with the procurement of Methadone and other supplies needed for seamless provision of OAT.

#### Establishment of OAT clinic

UMB collaborated with the MoH -NASCOP, CSOs, and Nairobi City County (NCC) to assess and identify a facility where OAT services could be provided. This paved the way for the opening of first OAT clinic in Kenya in December 2014 at Mathari National Teaching and Referral Hospital (MNTRH), in Nairobi County. MNTRH was selected as the first clinic as it is Kenya's national teaching and referral hospital offering psychiatric services and provides addiction treatment services. The facility had addiction management specialists who provided clinical support for OAT services. Suitable clinic space was identified, renovated, and equipped for the provision of comprehensive OAT services. The operationalization of this clinic required extensive microplanning, community engagement, and mobilization led by CSOs/CBOs. In February 2017, a second clinic was opened at Ngara Health Center in Nairobi to support scale- up of services. Ngara Health Center was chosen due to its proximity to high density hotspots for PWID and to CBOs/CSOs providing services in Drop-in Centers (DICs) within the facility's catchment area.

#### Infrastructure development

It was critical to identify good clinic space that would support the provision of comprehensive health services including triage, nursing procedures, HIV testing services (HTS), counseling, and psychosocial services. Further, there was need for patient observation room particularly following Methadone induction, clinical consultations and pharmacy for drug dispensing. There was also needed to secure room for Methadone storage, laboratory, health records storage and a data entry room, and a conference room for meetings and case discussions. Infrastructural adjustments were made to accommodate these requirements and were inspected and certified by the appropriate regulatory bodies, including PPB, KEMSA, and MoH. Security cameras were set up to ensure the safety of Methadone and other commodities and security of the HCPs working in the clinic. *MethaMeasure* was procured and installed in the pharmacy for Methadone dispensing.

### Developing competent healthcare providers

A multidisciplinary team of healthcare providers (HCPs) was identified and deployed to provide services in the OAT clinic. This included clinicians (doctors and clinical officers), nurses, laboratory technologists, pharmacists and pharmaceutical technologists, addiction counselors, health records and information officers, and social workers. This was supported by the health facility, MNTRH, the county department of health and supplemented by UMB- the implementing partner. The specific responsibilities of each cadre of the HCPs are shown in the Table 1.

**Table 1:** Roles and responsibilities of healthcare providers supporting OAT.

Cadre	Roles and responsibilities
Clinical officers	OAT induction and monitoring, dose adjustment, provision of clinical services including STI and TB screening and treatment of other illnesses, ART initiation and monitoring and mental health management.
Pharmacists	Methadone dispensing, dispensing of other drugs as needed including ARVs, commodity management and reporting and reporting of adverse drug reactions/pharmacovigilance
Nurses	Patient triage, HTS, basic counseling, and appointment management.
Addiction counselors	Provided psychosocial evaluation, prepared and implemented treatment plans, counseling, and support as well as client tracking
Laboratory technologists	Laboratory testing including HBSAg, HCV Ab, urine toxicology screen, blood sample collection for viral load monitoring.
Health records and information officers	File preparation, management, and storage, updating data on electronic medical records (EMR) platform, preparation, and submission of reports
Social workers	Link between community services and facility, client preparation in the DICs, follow-up of missed appointments, client tracking, and support for re-integration with families
Psychosocial counselors	Psychosocial assessment at OAT initiation, follow-up counseling, facilitate psychosocial support group meetings, preparation and review of OAT treatment plans and patient tracking
Security officers	Maintain a secure and safe environment, security checks, crowd management and maintain order in the waiting area

*STIs, sexually transmitted infections; DICs, drop-in centers; TB, tuberculosis; HTS, HIV testing services; ART, antiretroviral therapy; EMR, electronic medical records*.

HCPs were trained using the national curriculum and received continuous technical support through on-site mentorship by technical teams from NCC and UMB. Biweekly video conferences were held with the expert team (addiction psychiatrist, nurse, social worker) from the Department of Psychiatry at UMB in the United States for continuous capacity building.

### Client identification and recruitment

**Figure 1:**
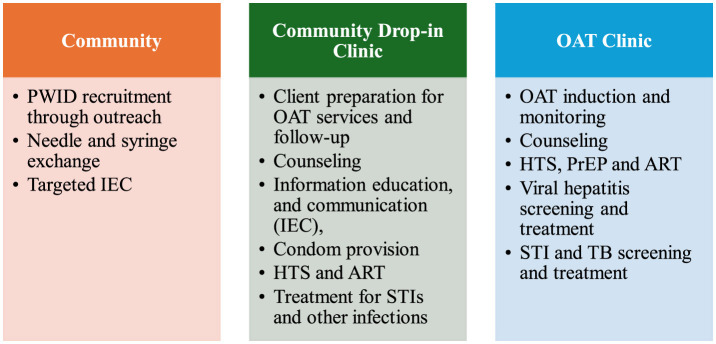
Services provided at each entry point from the community to OAT clinics.

Identification of PWIDs began at the community level during outreach activities by CBOs/CSOs in the hotspots (Figure 1). Clients were counseled and referred to DIC for enrolment and provision of health services including STI screening and treatment, HIV testing and ART initiation, and screening, counseling, and preparation for OAT, using structured tools. Those eligible for OAT had to have a definitive diagnosis of opioid use disorder, as per the Diagnostic and Statistical Manual of Mental Disorders, Fifth Edition (DSM-5) criteria (28) and were above the legal age of 18 years with the ability to provide consent for treatment. The original eligibility criteria restricted enrollment for individuals injecting heroin alone, but this was expanded to include individuals who smoked heroin. Preparation sessions were typically delivered over five sessions. Once an individual was ready to be initiated on OAT, they were escorted to the OAT clinic by CSO staff on a pre-agreed date to minimize attrition. To harmonize client enrolment from the three different CSOs/CBOs, the OAT clinic stipulated days/weeks pre-assigned to each organization, for client inductions. This ensured coordinated planning and preparation of clients for enrolment into the OAT clinic.

### OAT service delivery: clinic flow

**Figure 2:**
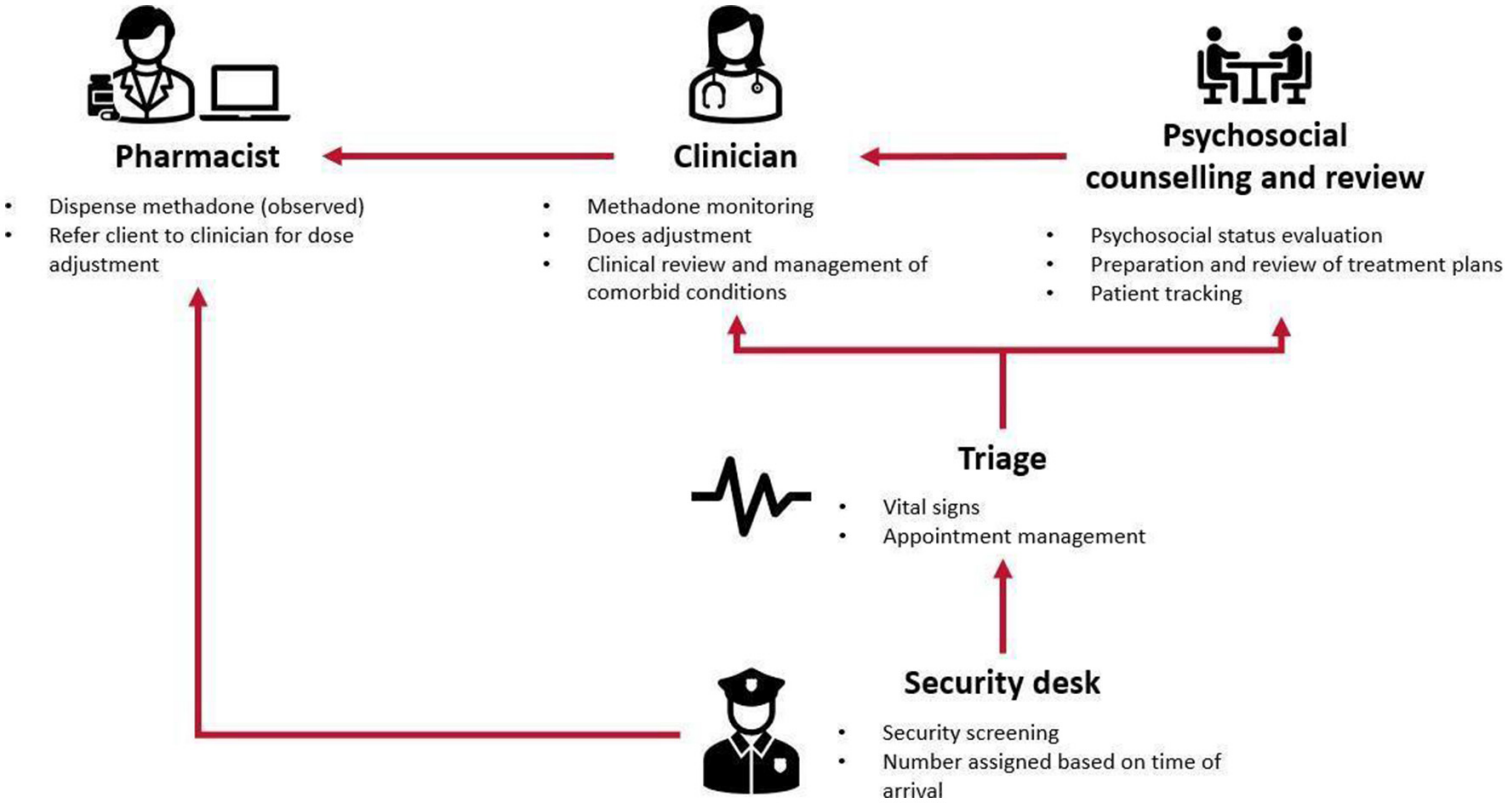
Flow of OAT clinic services.

In the OAT clinic, all clients underwent security screening at entry after which a card was given to each of them showing their position in the queue for crowd control (Figure 2). Services were offered on a first-come, first-served basis. Newly enrolled clients were escorted to the laboratory where urine drug testing and other baseline laboratory tests were conducted. The clients were then escorted to the data office where documents received from the CSO/CBOs were reviewed and their eligibility confirmed based on the national guidelines. The health records officer/data officer completed the initial registration form after which the clients proceeded to nursing for triaging, drug use history and assessment of withdrawals using the Clinical Opioid Withdrawal Scale. The clinicians conducted a comprehensive medical and mental health evaluation to confirm eligibility, after which the clients were escorted to the pharmacy for registration on *MethaMeasure* using their fingerprints for biometrics. Further, clients' photographs, were taken and their bio data captured specifically, their age, sex, date of enrollment and locator information. The clients received a psychosocial assessment and treatment planning. During subsequent visits clients were fast tracked Methadone dispensing to minimize time spent in the clinic. Clients booked for other services, including HTS, psychosocial counseling follow-up, and clinical follow- up including management of other comorbidities including HIV, TB, STI, hepatitis B and C, had to first receive these services during subsequent visits, prior to Methadone dispensing to minimize loss to follow up.

#### OAT dispensing

Methadone was dispensed as direct observed therapy in the pharmacy using *MethaMeasure* which utilizes biometrics to uniquely identify clients. *MethaMeasure* flags clients who are due for clinical review and/or dose adjustment. The dispensing area allowed for privacy through one-one-one interaction with the pharmacy staff who dispensed other medications needed by the clients. Methadone was dispensed to clients daily (including weekends and holidays). To allow this to happen, the clinics were open every day of the week, with clinics opening as early as 6:30 a.m. to allow for those in employment to get their treatment early. This model allowed daily observed therapy (DOT) of antiretrovirals for those on HIV treatment.

#### Urine toxicology screening

At treatment initiation, and quarterly thereafter, point-of-care immunoassay urine drug screening was conducted for every client, to monitor continued drug use. Results of urine drug screen were reviewed by the clinicians and addiction counselors to determine the need for OAT dose adjustment or for provision of psychosocial evaluation and support to identify and address factors associated with continued use of illicit drugs as needed. The recommended point-of-care immunoassay urine drug screening commonly tested for Methadone, heroin, benzodiazepines, barbiturates and amphetamines.

#### Data management

Routine client data was collected in national standardized forms at each encounter with the client. These tools included: 1) Initial registration form 2). OAT consent form 3). Clinic intake form 4). Psychosocial intake form 5). Clinic follow-up form 6). Psychosocial support follow-up form 7). Methadone induction and monitoring form 8). OAT dose request form 9). OAT random client monitoring form 10). Care ending and tracking form 11). HIV care client encounter form. In 2016, 2 years after inception of the OAT program, the tools used were revised to include community tools for OAT namely: CSO eligibility screening and referral form, CSO referral form, OAT daily activity register, CSO psychosocial support follow-up form, OAT clinic referral/transfer form, voluntary and involuntary discontinuation form, assessment checklist for voluntary cessation as well as pharmacy inventory, dispensing and reporting forms.

In 2018, UMB developed and deployed point of care electronic medical record (EMR) and dispensing system in both OAT clinics in all service delivery points. This process was built on work started by the ICAP at the Jaramogi Oginga Odinga Teaching and Referral Hospital Wellness Center and culminated in the development of the OAT module on the IQCare EMR platform. This platform contained all the national OAT data capture tools. Data reconstruction was done to transfer data from paper to the EMR, with data quality assessments showing >95% concordance between paper- based records, *MethaMeasure*, and EMR. Furthermore, software upgrades were conducted on the dispensing software *MethaMeasure* with the aim of system integration with IQCare to allow for generation of accurate data for reporting and streamlining service delivery between pharmacy and other clinical and psychosocial service delivery points. Following this, the facilities generated data on the progress of the OAT services, for decision making, to enhance clinical care and for routine reporting to the national program and to the donors. The program defined indicators to assess program performance and inform service delivery.

#### Technical support

UMB has been at the center of the opioid crisis since the 1960s and has been providing OAT since the early 1970s. Experts from the University of Maryland School of Medicine (UMSOM) Department of Psychiatry's Division of Addiction Research and Treatment including addiction-boarded psychiatrists, nurses and the social worker/clinical director provided training and mentorship to the Kenya-based staff.

#### Role of funding agency/CDC

Funding from PEPFAR through CDC supported the establishment of the clinic infrastructure, procurement and maintenance of equipment and consumables for the clinic, including CCTV, Methameasure, laboratory reagents and urine toxicology kits, hiring, training, and mentorship of additional HCPs to provide services in the OAT clinics and the deployment and maintenance of the EMR system. CDC participated in the development of policies and guidelines at national level, monitoring progress in the implementation of OAT services and facilitated the introduction of evidence-based strategies for better impact. Other agencies including UNODC and USAID played a critical role in the expansion of the OAT clinics to the coastal region and participated in the national task force that spearheading the expansion of services for PWID in Kenya.

#### Role of CSOs/CBOs

The CSOs/CBOs were instrumental in the mobilization of PWIDs for OAT services through outreaches in hotspots, They provided the harm reduction package of services in the community-based DICs, prepared clients for OAT services, linked them to the OAT clinic for initiation and support follow-up at community level, including support for treatment retention. Furthermore, as a critical link to the community, CSOs provided psychosocial support, including re-integration into society for clients who had been rehabilitated. They also served as support for the reengagement of individuals who had dropped out of treatment at the OAT program.

#### Expansion

To enhance access for OAT to PWID in Nairobi, and with upsurge of the COVID-19 pandemic in 2020, the Ministry of Health, NASCOP, UMB, Red Cross, and NCC, working through harm reduction Technical Working Group (TWG), designed and implemented a mobile OAT clinic to provide services in areas distant from the 2 static clinics. To structure this, the team developed mobile van dispensing SOPs, in collaboration with CSOs, which highlighted the mapping of routes and supervision of the processes.

#### Ethical review and approval

This community case study is based on an implementation protocol that was reviewed and approved by the Kenyatta National Hospital–University of Nairobi, Ethics and Scientific Review Committee [KNH-UON ERC (P435/06/2018)] and the University of Maryland Baltimore (UMB) Institutional Review Board (HP-00086720).

### Program achievements

Between 2014 and 2023, a total of 3,354 clients were inducted on OAT in the two clinics of Ngara and MNTRH, 90% of these were male clients, and more than 80 HCPs were trained on the provision of OAT services. At OAT induction, 98% of individuals received urine toxicology screening, 76% HIV testing, 73% Hepatitis B screening, 73% Hepatitis C screening, and 100% were screened for TB screening (Table 2). There were 222 individuals infected with HIV and of these, 193 (87%) were initiated on ART with 100% virally suppressed at 12 months of treatment. While there were shortages of testing kits for HIV, Hepatitis B and C testing between 2022 and 2023 period, STI and TB commodities remained available over the years. The OAT clinics engaged small-scale public-private partnerships with local well-wishers and the National Government Affirmative Action Fund to provide vocational training for those on treatment. This led to the establishment of self-help groups for patient empowerment to support re-integration of rehabilitated individuals into society.

**Table 2:** Enrolment in OAT Clinics and uptake of baseline screening tests 2014–2023.

Year	Newly enrolled in OAT			Urine drug screening		HIV testing		Hepatitis B screening		Hepatitis C screening		TB screening	
	Male	Female	Total	*N*	%	*N*	%	*N*	%	*N*	%		
2014	17 (77%)	5 (23%)	22	22	100%	16	73%	10	45%	10	45%	21	95%
2015	444 (84%)	87 (16%)	531	473	89%	340	64%	162	31%	165	31%	529	100%
2016	35 (83%)	7 (17%)	42	42	100%	42	100%	42	100%	42	100%	42	100%
2017	220 (90%)	25 (10%)	245	245	100%	245	100%	245	100%	245	100%	245	100%
2018	833 (93%)	62 (7%)	895	895	100%	895	100%	895	100%	895	100%	895	100%
2019	353 (92%)	31 (8%)	384	384	100%	384	100%	384	100%	384	100%	384	100%
2020	112 (91%)	11 (9%)	123	123	100%	123	100%	123	100%	123	100%	123	100%
2021	230 (92%)	19 (8%)	249	249	100%	249	100%	249	100%	249	100%	249	100%
2022	407 (91%)	41 (9%)	448	448	100%	95	21%	187	42%	188	42%	448	100%
2023	384 (93%)	31 (7%)	415	415	100%	158	38%	158	38%	158	38%	415	100%
Total	3,035 (90%)	319 (10%)	3,354	3,296	98%	2,547	76%	2,455	73%	2,459	73%	3,351	100%

## Discussion

The purpose of this paper was to describe the pathway to establishing and implementing OAT services in Kenya based on experiences from MNTRH and Ngara Health Center in Nairobi where the first clinics were established in 2014. The OAT services were initiated under the leadership of the MoH through NASCOP, in close collaboration with the UMB, NCC, CDC, CSO and CBOs working with PWID, and other key stakeholders. Its introduction was premised on the need to reduce HIV acquisition and transmission among PWID. The package of care went beyond drug dependence treatment to include the prevention and treatment of HIV, TB, STI, viral hepatitis as well as provision of IEC materials and psychosocial support to OAT clients.

The roll-out of the OAT program was critical in not only supporting the government's efforts to reduce HIV incidence in PWID but also necessary to optimize treatment outcomes among those who are HIV infected. Studies have shown that PWID who were not enrolled in OAT program were threefold more likely to be off ART and twice as likely to be viremic compared to those in receiving OAT (29). Several countries in SSA have experienced delays in introducing harm reduction programs targeting PWID, with several countries in Eastern and Southern Africa continuing to have repressive criminal laws, high estimated transmission rates of HIV and viral hepatitis among PWID. Further, the absence of essential health services has hampered implementation of harm reduction services in the region (20).

Following the implementation of OAT services over 10 years, notable achievements include: development and roll-out of the first national guideline for OAT which has subsequently been revised to accommodate other OAT drugs and dispensing models; 2) Development of national training curriculum on OAT, which has led to the training of many HCP countrywide resulting in the production of a critical mass of providers skilled in OAT services; 3) Expansion of clinics providing OAT from 2 to 10 clinics in the 4 high burden counties by 2024; 4) Evolution of OAT dispensing from static to the current hybrid of static and mobile dispensing leading to increased coverage and continuity in treatment; 5) Strong linkage between the OAT clinics and the community, supported by CBOs/CSOs and; 6) Enhanced collaboration with stakeholders including the criminal justice system which has reduced stigma associated with opioid use and ensured continued access to OAT for clients in police custody.

Several lessons can be learned from Kenya's journey in rolling out OAT services and should be considered as countries establish these services. These include: 1) OAT clinics should be situated near PWIDs for enhanced access to the services. The number of clinics in a region should be informed by the mapped population of PWID. There are still only two static OAT clinics in Nairobi, a county with 73 mapped hotspots and an estimated 5,024 PWID (32); hence, there is need for an expansion strategy for increased coverage. 2) Daily methadone dosing as per the current drug policy in Kenya presents a major barrier to treatment retention, leading to high patient attrition rates. Patient dropouts escalate when individuals travel to regions lacking Opioid Agonist Treatment (OAT) clinics or face inflexible working hours. Furthermore, clinic access is routinely compromised by prohibitive commuting costs, healthcare provider strikes, medication shortages, social unrest, and pandemic-related travel restrictions as was the case for COVID-19. Adopting take home dosing, mobile dispensing, use of OAT formulations that do not require frequent clinic visits and other person-centered approaches, could address these challenges and the inconvenience of daily clinic visits.

Though Kenya is yet to implement take-home doses, there are provisions for this in the guidelines updated in 2022. Take home doses can be implemented in the event of illness that interferes with ability to come to the clinic daily, personal or family crisis, travel, pregnancy or post-partum care, clients in need of split dosing, religious practices (e.g., Ramadhan), disasters and civil/political unrest and other hardship that interfere with daily dosing at the clinic. Other strategies that have been employed include engagement of law enforcement to support movement during unrest, task shifting of service provision during staff shortages, and mobile Methadone dispensing. The updated 2022 guidelines have also expanded formulations including Buprenorphine and Naltrexone. This is in keeping with the global increase in the use of long-acting injectable forms of Buprenorphine (weekly and monthly formulations) and Naltrexone (monthly formulation).

To continue expanding treatment coverage, programming efforts should prioritize reaching opioid users who remain underserved. Women who use drugs are a key population of concern because their enrolment in OAT clinics nationwide remains low compared with men (10 vs. 90%, respectively). Targeted strategies are therefore needed to increase uptake of OAT services among women. Given the stigma associated with opioid use, surveys among women who use opioids are needed to generate evidence that can inform tailored interventions to improve coverage. Studies also highlight the absence of formal harm reduction programs for stimulant use and new psychoactive substances in Eastern and Southern Africa, an area requiring further attention (20). Sustainability of OAT clinics beyond PEPFAR funding is another key concern, as OAT is a critical and cost-effective service (30). Although Kenya's national and county governments have demonstrated strong ownership and leadership of the program, OAT services remain donor funded. Dedicated budgeting for OAT programs in Kenya and other low- and middle- income countries is urgently needed to sustain this essential service and support progress toward HIV epidemic control.

## Conclusion

The journey toward the establishment of OAT services in Kenya started in 2013 to address increasing opioid use, resulting in the successful opening of the first OAT clinic in 2014. Over the past decade, services have been expanded to 10 clinics in four high burden counties. Innovative practices, such as mobile OAT dispensing vans, have allowed clinics to expand coverage and improve continuity of treatment. Moving forward, the future of OAT in Kenya may include the adoption of take-home doses of OAT using Methadone or Buprenorphine, expanded mobile services, expanded satellite dispensing sites (including pharmacies, DICs and correctional facilities), and adopting injectable long-acting buprenorphine.

## Data Availability

The raw data supporting the conclusions of this article will be made available by the authors, without undue reservation.
